# Effectiveness of Transitional Care Interventions for Heart Failure Patients: A Systematic Review With Meta-Analysis

**DOI:** 10.7759/cureus.29726

**Published:** 2022-09-29

**Authors:** Aya Al Sattouf, Rasha Farahat, Aayesha A Khatri

**Affiliations:** 1 Medicine, West Suffolk NHS (National Health Service) Foundation Trust, Suffolk, GBR; 2 Haematology, King's College NHS (National Health Service) Foundation Trust, London, GBR

**Keywords:** telephone-based support, telemonitoring, effectiveness, hospital re-admission, heart failure, transitional care interventions

## Abstract

Heart failure is a leading cause of hospitalizations. Heart failure patients were found to have a high incidence of re-admission after discharge. This highlights a care gap during the transition from hospital to home environment and interventions were utilized to cover this care gap. The aim of this review was to evaluate the effectiveness of these interventions. This was investigated in terms of re-admissions, mortality, emergency department (ED) visits, and quality of life.

An exhaustive systematic search was conducted in electronic databases, which include MEDLINE, CINAHL, AMED, Cochrane library, and PubMed. Databases were explored for literature published in English between April 2012 and April 2022. The review included 13 randomized controlled trials and comprised a total of 7,693 heart failure patients with 3,835 receiving transitional care interventions (TCIs) and 3,858 receiving standard care. It was found that implementing TCIs resulted in a reduction of all-cause re-admission and all-cause mortality. Although it is controversial if TCIs improve quality of life, TCIs were noted to decrease the frequency of ED visits. Telephone support interventions proved most efficacious among other interventions in reducing hospital readmissions, and were found effective in reducing mortality in combination with other interventions, i.e. clinic visits. Additionally, telemonitoring is found beneficial in supporting patients just after discharge, the most vulnerable period, for medically optimizing and monitoring patients during the care gap.

## Introduction and background

Heart failure (HF) was classified as a global pandemic by the British Heart Foundation (BHF) [1]. About 64.3 million people worldwide suffer from HF [2]; it was estimated that HF affects about 1-2% of the population in developed regions [3]. This estimate is based on data reported in registries and lacks consideration for undiagnosed cases in the community. It is argued that true prevalence is much higher due to the high number of undiagnosed cases. A study that used echocardiography screening for HF reported a prevalence of 11.8% among the population aged 65 and above in developed countries [4]. The prevalence of HF increases gradually up until the age of 65 before accelerating after that age [5]. As opposed to one in 35 persons between the ages of 65 and 74, it was predicted that slightly over one in seven people aged 85 and older will have HF [5].

A diagnosis of HF carries a significant financial burden on both the economy and the individual. This is explicable given the substantial morbidity and mortality linked to HF. Fifty percent of patients diagnosed with HF die within five years after their initial diagnosis [5]. Years of life lost (YLL) due to HF is 9.64 years, which is significantly higher than YLL due to dementia and osteoporosis [6]. An average of 1.1 to 2.3 years are lost by HF patients due to early death or disability [7]. It is reported that HF-related costs account for 1-2% of the overall budget for healthcare systems [5]. The estimated yearly cost of HF is $108 billion, with direct-related costs accounting for $65 billion [8]. It is projected that HF treatment would cost an individual about $24,383 per year [9], which is a 318% rise compared to medical expenses in the year proceeding diagnosis [10].

HF is one of the common reasons leading to hospitalization in those 65 and older and is considered a predictive factor of higher mortality [11]. During the initial six months after discharge, one-half of HF patients are readmitted and up to one-fourth are readmitted within 30 days of discharge [12]. Hospital readmission is known to cause a significant burden on the healthcare system and the individual. Therefore, in order to decrease the frequency of readmission, it is crucial to comprehend and address the underlying causes. Inadequate patient education, lack of identification of symptoms requiring medical attention, and poor compliance with medications and lifestyle modifications are among the commonly reported causes of increased readmission [13]. This reveals a discontinuity in the care given to patients when they move from the hospital to their homes. According to reports, one-fourth of HF readmissions can be prevented [12]. Therefore, significant effort was implemented to study interventions that would reduce hospital readmission among HF patients. Additionally, governments have also contributed to these efforts. For example, Congress reduced hospitals' yearly Medicare reimbursements by up to 3% if their readmission rates were higher than average [14].

Transitional care interventions (TCI) are founded to ensure continuity and coordinated care provided to HF patients transitioning from hospital to place of usual residence [15], which cover the gap created by this transition. TCI includes pre-discharge and post-discharge interventions, which include proper education and effective communication of instruction, medication explanation, and post-discharge follow-up to assess self-management [16]. TCIs can be classified into categories, including telemonitoring, home visits, outpatient clinic intervention, structured telephone support, and educational interventions [16]. Although many randomized controlled trials (RCTs) were done to study the effectiveness of TCIs, this remains debatable as some agreed on the effectiveness of TCI [17], and others reported that TCI did not reduce hospital readmission [18]. Due to this controversy and the significant benefit obtained from implementing TCIs, it was worth investigating its effect on hospital readmissions, emergency department (ED) visits, mortality rates, and quality of life (QoL). Primary research, such as RCTs, is usually used to study the effectiveness of an intervention. However, the number of primary studies in the literature can be overwhelming and this makes clinical decision-making challenging. Moreover, most of the previous reviews do not reflect recent TCIs studied in the current literature and are thus out of date [19]. Therefore, a systematic review with meta-analysis was done with an aim to generate an up-to-date summary with statistical analysis to inform the decision-making of healthcare practitioners.

## Review

Methodology

The Preferred Reporting Items for Systematic Reviews and Meta-Analyses (PRISMA) recommendations were followed when conducting this systematic review and meta-analysis [20], and steps recommended by the Cochrane Handbook of Systematic Reviews for Interventions were followed throughout this review [21].

Literature Search

To ensure consistency and transparency throughout the review and reduction of bias, a protocol for systematic review was designed prior to the commencement of the review [20]. MEDLINE, CINAHL (Cumulative Index to Nursing and Allied Health Literature), AMED (Allied and Complementary Medicine Database), Cochrane library, and PubMed were extensively and systematically searched for relevant most recent literature available in English between April 2012 and April 2022. It was found that the greatest hierarchy in determining the efficacy of an intervention is by reviewing RCTs due to bias minimization by giving direct comparisons to the intervention under study [20]. Thus, a search was limited to RCTs only. Key terms used to search for relevant studies include effectiveness, chronic heart failure, and transitional care interventions. In addition, synonyms, types of TCIs, and outcomes studied were also used. (Heart failure AND telemonitoring AND effectiveness), (heart failure AND clinic-based follow-up AND readmission), (heart failure AND home visit programs AND effectiveness), (heart failure AND Structured telephone support AND readmission), (Heart failure AND transitional care interventions AND readmission OR mortality), and (Chronic heart failure AND patient education AND readmission) are examples of effective combinations.

Inclusion and Exclusion Criteria

PICOTS acronym was used to develop strict exclusion and inclusion criteria for the systematic review [21] (Table 1), which stand for Population, Intervention, Comparison, Outcome, Timing, and Study design of included studies.

**Table 1:** Inclusion and exclusion criteria HF: heart failure; TCIs: transitional care interventions; ED: emergency department; QoL: quality of life; RCTs: randomized controlled trials.

	Inclusion criteria	Exclusion criteria
Population	Adults who are 18 years and older and had HF needed hospitalization. Subjects enrolled in the trial during or soon after an index hospitalization for HF-related illness.	Patients hospitalized for reasons unrelated to HF. Children and subjects aged less than 18 years.
Intervention	TCIs are being introduced among HF patients in order to prevent hospital readmissions, and include any of the following: home-visit programs, structured telephone support, telemonitoring, clinic-based interventions, primarily educational interventions.	Surgical, pharmacological, and invasive interventions implemented for HF management.
Comparison	Usual care	Studies that compare intervention with another intervention.
Outcome	Trials that measure mentioned outcomes of intervention: Primary outcome: hospital readmission. Secondary outcomes: mortality rates, ED visits, and QoL.	Trials that study cost-effectiveness, or report outcomes other than those measured in this review.
Timing of measured outcome, follow-up length	Trials that measure the outcomes within six-month period following an index hospitalization related to HF. Follow-up lasting for 30 days or more.	Outcome measured beyond the six-month period. Follow-up lasting less than 30 days.
Time period	Trials conducted within the last 10 years, i.e., April 2012 to April 2022.	Studies that do not meet the time scale.
Settings	Interventions are implemented in an inpatient setting or shortly after discharge following an index hospitalization with an aim of facilitating the transition from hospital to home.	Interventions implemented in rehabilitation or nursing care facility.
Language, peer-review, ethical approval	English, peer review, studies with confirmed ethical approval.	other languages, no peer review, no ethical approval.
Study types	RCTs.	Other research design.

Data Selection

Retrieved literature was screened initially by reading the title and abstract and irrelevant trials were eliminated. Subsequently, studies were assessed by reading their full text to assess their eligibility for inclusion. The review included only studies that fully complied with inclusion criteria. EPPI-Reviewer software was used to facilitate data selection and management.

Data Extraction

Data from selected RCTs were electronically extracted using a form created with the Microsoft Office Excel program. Information about the baseline characteristics of RCTs included in the review was recorded in the extraction forms and included the sample size and average age, predominant gender, and HF severity of participants included. In addition, information about the duration of RCTs, intervention studied, delivering personnel, and reported outcomes were also extracted.

Data Analysis

Meta-analysis was conducted to investigate the efficacy of TCIs in lowering ED visits, readmissions, and mortality while also improving patients' QoL. Due to the variability of TCIs, the statistical analysis was stratified by intervention type and timing of outcome measurement of both readmission and mortality into those occurring within 30 days and beyond 30 days of discharge.

Review Manager (RevMan 5.4) software was utilized to conduct the statistical analysis for this review. To determine the pooled effect size, a random-effect model was used because included RCTs are not completely identical. For binary outcomes, such as mortality and readmission, the pooled effect was calculated using relative risk (RR) with a 95% confidence interval (CI). In contrast, standard mean difference (SMD) was used to calculate the pooled effect for continuous outcomes, such as QoL. The pooled analysis was graphically represented using forest plots.

Heterogeneity among studies included in the analysis was estimated by calculating chi-square and I^2^ statistics. I^2^ value >75% indicates a considerable heterogeneity. Methodological and clinical diversity was explored to rationalize significant heterogeneity, when found, among studies included. To assess the generalizability and applicability of findings, the strength of evidence (SOE) was evaluated. The GRADE approach was used, which entails an organized and transparent appraisal of the results against the domains, risk of bias, indirectness, inconsistency, imprecision, and publication bias [21]. SOE can be classified as high, moderate, low, or very low.

Risk-of-Bias Assessment

Although randomization is used in RCTs, bias is still a major problem. Cochrane Handbook for Systematic Reviews recommended using the Risk of Bias 2.0 (RoB 2.0) tool for quality assessment of included RCTs [21]. It is a well-structured tool to evaluate the risk of different types of bias, which include selection bias, performance bias, detection bias, attrition bias, and reporting bias. The ranking system will be used to categorize each study's likelihood of bias as low, some concern, or high. Studies are deemed valid when there is a low risk of bias. Studies that received some concerns are also regarded as having a significant chance of bias, but not enough to render the study invalid. However, studies with a high risk of bias indicate significant flaws in the study's design and were disregarded.

Results

Results of Literature Search

**Figure 1:**
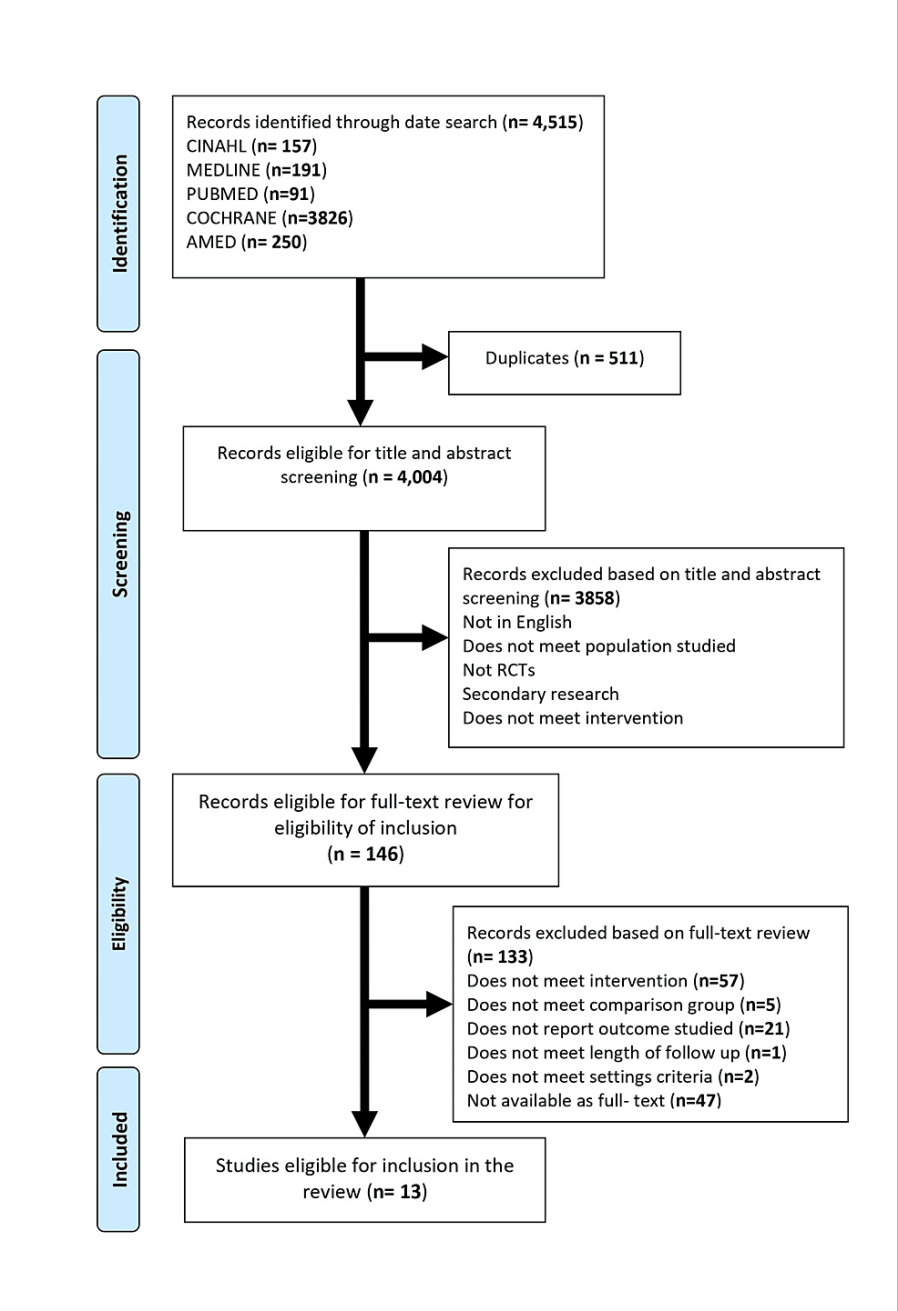
PRISMA flow diagram of the literature search. PRISMA: Preferred Reporting Items for Systematic Reviews and Meta-analyses; CINAHL: Cumulative Index to Nursing and Allied Health Literature; AMED: Allied and Complementary Medicine Database; RCTs: randomized controlled trials.

Literature search and study selection are explained in the PRISMA flow diagram, as shown in Figure 1. A thorough search of the databases retrieved 4,515 articles, 3,858 of them were eliminated after being screened by title and abstract, and then, 146 RCTs underwent full-text screening. After careful evaluation of research design, intervention stated outcomes, and author credibility, 13 studies were included in the review.

Baseline Characteristics of Included RCTs

Thirteen RCTs that examine the effectiveness of different TCIs were included in this study. The review included 7,693 patients with HF, 3,835 of whom received TCIs and 3,858 of whom received standard care. Participants' average ages ranged from 50 to 85 years. Male participants made up between 14% and 73% of the total population. The majority of individuals fell into New York Heart Association (NYHA) classes III-IV and had moderate to severe HF symptoms. Table 2 shows baseline characteristics of included RCTs.

**Table 2:** Baseline characteristics of included RCTs. NYHA: New York Heart Association Functional Classification; HF: heart failure; IHD: ischemic heart disease; RCT: randomized clinical trial.

Author name, year, and locale	Type of intervention and delivering personnel	Duration of follow-up and timing of measurement	Intervention (n) versus control (n)	Mean age, gender	Baseline severity of HF	Predominant comorbidities	Outcomes
González-Guerrero et al., 2014 [22], Spain, single center	Clinic-based (with telephone follow-up); multidisciplinary team	12 months. Outcomes measured at the end of follow-up period	Intervention (59) and usual care (58)	85 years, female (73%)	NYHA II or III, 47% or 38.5%, respectively	Hypertensive cardiopathy (45.3%), ischemic cardiopathy (27.4%)	All-cause and HF- specific readmission or death. Quality of life
Yu et al., 2015 [23], Hong Kong, single center	Home visit (with telephone support); nurse	9 months. Outcomes measured at 6 weeks, 3 months, 9 months	Intervention (90), usual care (88)	78.6 years, male (53.3%)	NYHA II (58.9%), III (37.8%), IV (3.3%)	Hypertension (66.7%), diabetes (40%), atrial fibrillation (28.9%), IHD (18.9%)	All-cause mortality and readmission. Quality of life
Vinluan et al., 2015 [24], USA, single center	Telephone follow-up (with predischarge counseling); pharmacist	3 months. Outcomes measured at 30 days and 2 and 3 months	Intervention (7), usual care (9)	74 years, female (86%)	NYHA not reported	Hypertension (100%), diabetes (57%), myocardial infarction (14%), renal failure (29%)	Medications adherence. Readmission. Mortality
Ong et al., 2016 [25], USA, multicenter	Telemonitoring (with predischarge education and regular telephone follow-up), nurse	6 months. Outcomes measured at 30 days and 6 months	Intervention (715) and usual care (722)	73 years, female (46.2%)	NYHA III or VI (61.2%)	Hypertension (81.7%), renal failure (39%), diabetes (44.8%), chronic pulmonary disease (32.4%)	30-day and 6-month all-cause readmission, 30-day and 6-month all-cause mortality, 3-day and 6-month quality of life
Boyde et al., 2017 [26], Australia, single center	Multimedia educational intervention, HF nurse	12 months. Outcomes measured at 28 days, 3 months, and 12 months	Intervention (100) and control (100)	64 years, males 73%	NYHA II-IV, majority are NYHA III (60%)	Myocardial infarction (55%)	All-cause HF-related readmission at 28 days, 3 months, and 12 months. Self-care knowledge
Frederix et al., 2018 [27], Belgium, multicenter	Telemonitoring (with telephonic follow-up), HF nurse	79 months, outcomes measured at 6 months and 79 months	Intervention (80), control (80)	76 years, males (64%)	Majority were NYHA class III	Comorbidities not reported	All-cause mortality. Number of days lost due to HF or all-cause readmission. Health-related cost
Huynh et al., 2019 [28], Australia, multicenter	Multicomponent (discharge timing optimization, education to improve self-care, liaising with primary care upon hospital discharge, and post-discharge surveillance using phone calls and visits to respond to instability). HF nurse or cardiologist	3 months. Outcomes reported at 30 days and 3 months	Intervention (215), usual care (197)	75 years, males (58%)	NYHA III-IV (70%)	Atrial fibrillation (50%), renal failure (36%), diabetes (37%)	30 days and 3 months mortality or readmission
Chen et al., 2018 [29], China, single center	Telephone-based intervention (structured telephone support vs. short message service), nurse	6 months. Outcomes measured at 6 months	Structured telephone support (255), short message service (252), usual care (260)	61 years, male (56%)	NYHA III-IV (68.4%)	Hypertension (32-36%), diabetes (27-30%), ischemic heart disease (18-22%)	Mortality and readmission at 6 months. Quality of life. Self-care behaviors.
Van Spall et al., 2019 [30], Canada, multicenter	Educational intervention (with primary care physician visit within 1 week. In addition, home visit and heart function clinic follow-up for high-risk patients). Nurse	3 months. Outcomes measured at 30 days and 3 months	Intervention (1104), usual care (1390)	77.7 years, female (50.4%)	NYHA not reported	Hypertension (71.3%), diabetes (70%), renal failure (22%), atrial fibrillation (53%)	All-cause readmission ED visit and death at 3 months. All-cause readmission and ED visit at 30 days. Quality of life. Discharge preparedness.
You et al., 2020 [31], China, single center	Structured telephone support, nurse	3 months. Outcomes measured at 3 months.	Intervention (80) vs. usual care (72)	50 years, male (73%)	NYHA III-IV (57%)	Hypertension (71%), dyslipidemia (66%), diabetes (58%), ischemic heart disease (61%)	Readmission and all-cause mortality. Quality of life.
Deek et al., 2020 [32], Lebanon, multicenter	Educational intervention (with telephone follow-up), delivering person not reported	12 months. Outcomes measured at 6 and 12 months.	Intervention (128) and usual care (132)	67 years, male (59%)	NYHA III-IV (33%)	Diabetes (46%), hypertension (73%), hypercholesterolemia (50%), ischemic heart disease (57%)	30-day readmission and mortality. Readmission and mortality at 6 and 12 months.
Kazemi Majd et al., 2021 [33], Iran, single center	Mainly educational (information prescription tolerated to patient needs), cardiologist and librarians	12 months. Outcomes collected at 6 and 12 months.	Intervention (60) and usual care (60)	66.5 years, male (40%)	NYHA III-IV	Diabetes (11%), asthma (3.3%)	Readmission at 6 and 12 months
Dawson et al., 2021 [34], USA, multicenter	Telemonitoring. Nurse	30-day outcome measured at 30 days	Intervention (690), usual care (690).	66 years, male (52%)	Not reported	Myocardial infarction (12%), diabetes (26%), renal disease (35.5%)	Readmission or death at 30 days. ED visit at 30 days

Risk-of-Bias Assessment

**Figure 2:**
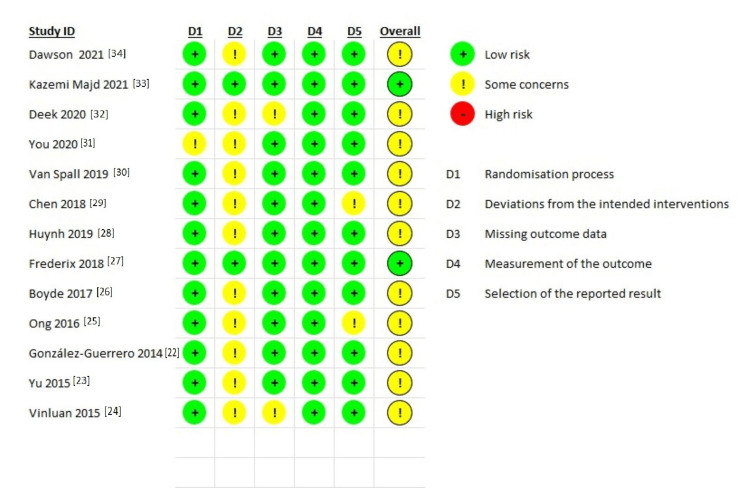
Risk-of-bias assessment

Most of the studies scored “some concerns” in risk-of-bias assessment except two studies scored low risk [33,27], as explained in Figure 2.

The process of randomization was acceptable among most of the studies included and ranging from computer-generated number to concealed envelopes. However, randomization was felt to be inadequate in one of the studies included [31], as allocation was decided by the last digit of the participants’ telephone numbers. However, all included RCTs did not show a significant difference in baseline characteristics among participants, which ensure adequate randomization.

The majority of included RCTs utilized a pretty optimal sample size. However, Van Spall et al. [30] used a large sample size. This increases the confidence in the effect estimate, even though it may result in higher costs and as well as ethical considerations due to a higher number of participants in the control group do not receive the intervention. In contrast, Vinluan et al. [24] used a small size, which had an impact on the reliability of the findings and raised the likelihood of type II errors.

In the section related to deviation from the intended interventions, the majority of the studies scored "some concerns" in the RoB assessment. This is mostly because of generally alarming dropout rates. This can be explained by the intervention design which hinders participants’ and delivering staff blinding. However, the majority of the studies used intention-to-treat analysis, which maintained the advantage of randomized allocation and provide unbiased results.

Effect of TCIs on Hospital Readmissions

**Figure 3:**
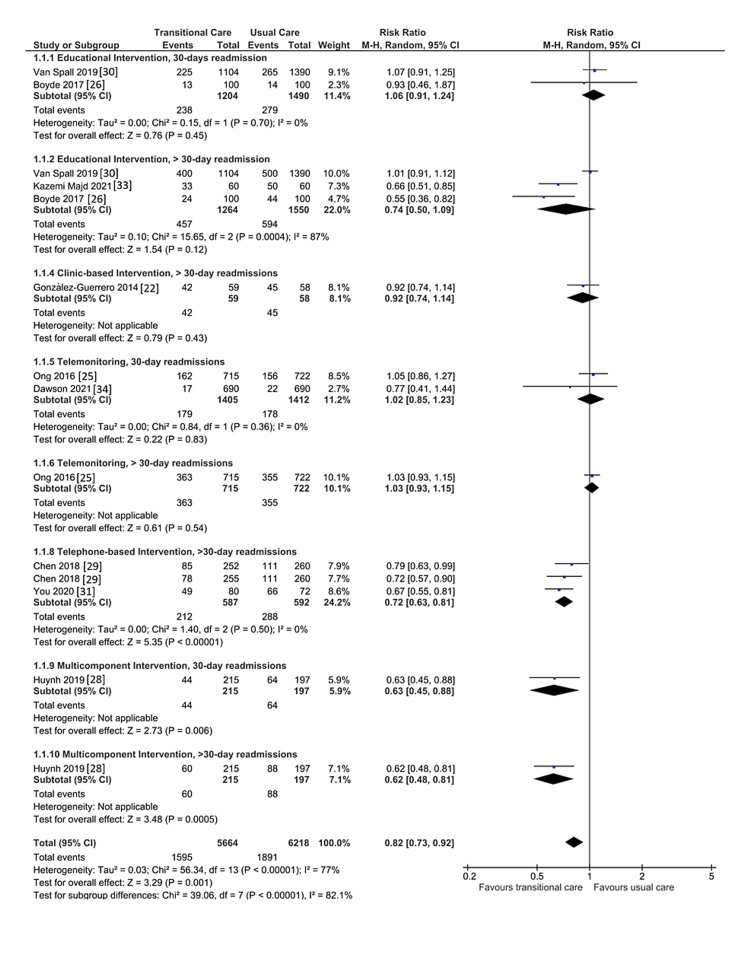
Forest plot showing the effect of TCIs on hospital readmissions CI: confidence interval; M-H: Mantel-Haenszel; TCI: transitional care intervention.

A meta-analysis was done to investigate the effect of TCIs on hospital readmissions, as shown in Figure 3. Four studies investigated the efficacy of educational interventions [26,30,32,33]. However, Deek et al. [32] reported the number of hospital readmission among intervention and control groups combined rather than separated and, therefore, was excluded from the meta-analysis. Education-based interventions, which was studied in two RCTs, were shown to be ineffective in reducing hospital readmissions within 30 days after discharge (RR, 1.06; 95% CI 0.91 to 1.24, p = 0.45). This analysis showed no significant heterogeneity (I^2 ^= 0%). Similarly, educational interventions did not lower hospital readmission beyond 30 days of discharge (RR, 0.74; 95% CI 0.50 to 1.09, p = 0.12), with significant heterogeneity (I^2 ^= 87%, p = 0.0004). This can be explained by variability in educational interventions among included RCTs. Educational interventions were supplemented by additional interventions, such as telephone support [32], multimedia delivering methods [26], follow-up by the general practitioners and home visits to high-risk patients [30], or education tailoring to patient needs [33].

The efficacy of home visit intervention cannot be ascertained as Yu et al. [23] reported percentages of participants in the form of a survival analysis for those who were not readmitted throughout the study. Therefore, the study was not included in the meta-analysis. However, the study reported that home-visit intervention was not effective in reducing hospital readmissions. Similarly, the meta-analysis revealed that clinic-based intervention [22] had no statistically significant effect on decreasing hospital readmission in the period beyond 30 days after discharge (RR, 0.92; 95% CI 0.74 to 1.14; p = 0.43).

This review included three RCTs that investigated the effectiveness of the telemonitoring intervention [25,27,34]. Fredrix et al.'s [27] were disqualified from the meta-analysis since they reported the number of days lost owing to hospital readmission rather than the actual number of readmissions. The pooled trials' statistical analysis showed that the telemonitoring intervention had no statistically significant effect on lowering hospital readmissions within 30 days of discharge (RR, 1.02; 95% CI, 0.85 to 1.23; p = 0.83) or afterward (RR, 1.03; 95% CI 0.93 to 1.15; p = 0.54).

Three trials studied the efficacy of telephone-based intervention [24,29,31]. Vinluan and colleague [24] carried out a comprehensive and organized telephone follow-up on the 3rd, 30th, 60th, and 90th days following discharge and was effective in reducing admissions within 30 days after discharge. However, this study was excluded due to the small sample size. Similarly, a meta-analysis found that telephone-based intervention was effective in reducing hospital readmission beyond 30 days after discharge (RR, 0.72; 95% CI 0.63 to 0.81, p = 0.00001). Similarly, hospital readmission within 30 days (RR, 0.63; 95% CI 0.45 to 0.88; p = 0.006) and after 30 days (RR, 0.62; 95% CI 0.48 to 0.81; p = 0.0005) was significantly decreased by multicomponent intervention.

Effect of TCI on Mortality Outcome

**Figure 4:**
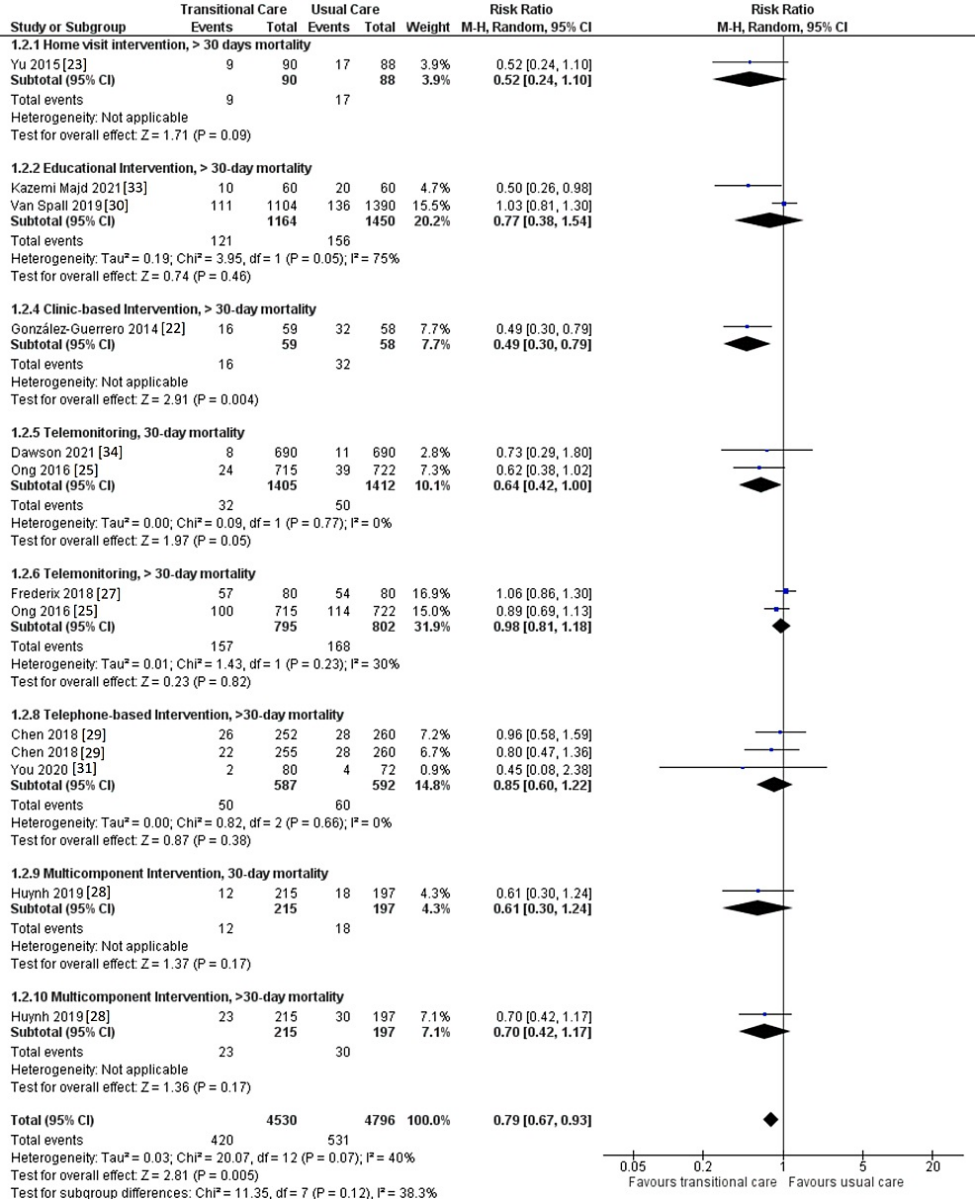
Forest plot showing the effect of TCIs on mortality CI: confidence interval; M-H: Mantel-Haenszel; TCI: transitional care intervention.

Figure 4 shows the meta-analysis for studies that reported mortality in their outcomes. Deek et al. [32] and Vinluan et al. [24] were excluded from the meta-analysis due to previously mentioned causes. In addition, the study conducted by Boyde and colleagues [26] was also excluded due to a lack of data on mortality outcome. It was found that TCIs were effective in reducing mortality in general (RR, 0.79; 95% CI 0.67 to 0.93, p = 0.005), which would favor their implementation. Heterogeneity among included studies is not significant as evidenced by the I^2^ value for overall analysis.

After 30 days of discharge, no discernible decrease in mortality was seen among those who underwent home visit (RR, 0.52; 95% CI 0.24 to 1.10, p = 0.09), educational intervention (RR, 0.77; 95% CI 0.38 to 1.54, p = 0.46), or telemonitoring intervention (RR, 0.98; 95% CI 0.81 to 1.18, p = 0.83). However, patients who received clinic-based care witnessed a reduction in mortality within the same time period (RR, 0.49; 95% CI 0.30 to 0.79; p = 0.004).

Despite the fact that these TCIs were effective in reducing readmissions, telephone-based intervention (RR, 0.85; 95% CI 0.60 to 1.22; p = 0.38) and multicomponent intervention (RR, 0.70; 95% CI 0.42 to 1.17; p = 0.17) were not effective in mortality reduction after 30 days for telephone-based intervention and within and after 30 days for multicomponent intervention. In contrary, telemonitoring interventions were effective in reducing mortality within 30 days (RR, 0.64; 95% CI 0.42 to 1.00, p = 0.05).

Effect of TCI on ED Visits 

**Figure 5:**
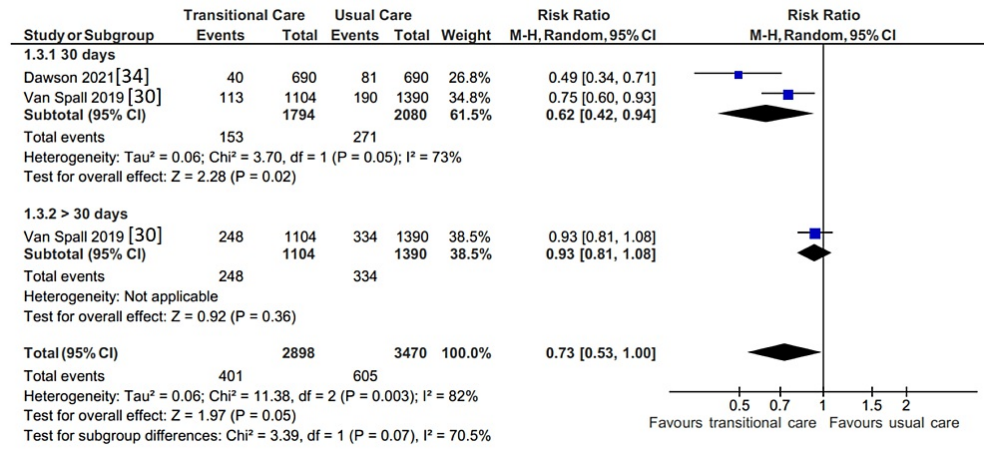
Forest plot showing the effect of TCIs on ED visits. CI: confidence interval; M-H: Mantel-Haenszel; TCI: transitional care intervention; ED: emergency department.

ED visits were recorded as a result of the telemonitoring intervention by two RCTs [30,34], and as a result, they were both included in the meta-analysis depicted in Figure 5. A decrease in ED visits within 30 days of discharge has been concluded (RR, 0.62; 95% CI 0.42 to 0.94, p = 0.02). However, it was discovered that the intervention was unsuccessful in lowering ED visits 30 days after discharge (RR, 0.93; 95% CI 0.81 to 1.08, p = 0.36). The substantial heterogeneity (I^2 ^= 73%) between the studies included is attributable to differences in the method and timing of follow-up. For example, Van Spall and colleagues [30] arranged for primary care physician follow-up within one week of discharge, early home visits, and weekly phone calls by the nursing team. In contrast, Dawson and colleagues [34] arranged home visits 72 hours after discharge to install equipment; the follow-up was limited during the study to only when alerted to an abnormality by the telemonitoring device.

Effect of TCI on QoL

**Figure 6:**
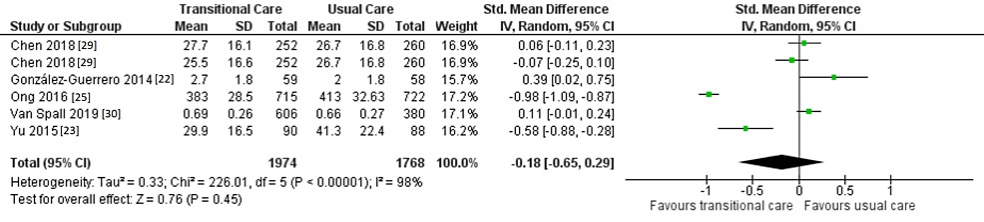
Forest plot showing the effect of TCIs on QoL CI: confidence interval; IV: inverse variance; SD: standard deviation; TCI: transitional care intervention; QoL: quality of life.

Six studies that evaluated the effect of TCI on QoL beyond 30 days following hospital discharge were included in this evaluation except for a study that did not report the mean and standard deviation for QoL assessment [31], as illustrated in Figure 6.

The statistical analysis of the effect estimates revealed that TCI did not enhance patients' QoL (SMD -0.18; 95% CI -0.65 to 0.29, p = 0.45). Chen et al. [29], González-Guerrero et al. [22], and Van Spall et al. [30] presented data that were in line with the overall analysis, suggesting that telephone assistance, educational interventions, and clinic support were ineffective at enhancing QoL. In contrast, Ong and colleagues [25] and Yu and colleagues [23] found that telemonitoring and home visits, respectively, can both enhance QoL. Telemonitoring had the best results in terms of QoL (SMD -0.98, 95% CI -1.09 to -0.87). This might be attributed to frequent phone coaching and predischarge education offered to RCT participants.

**Figure 7:**
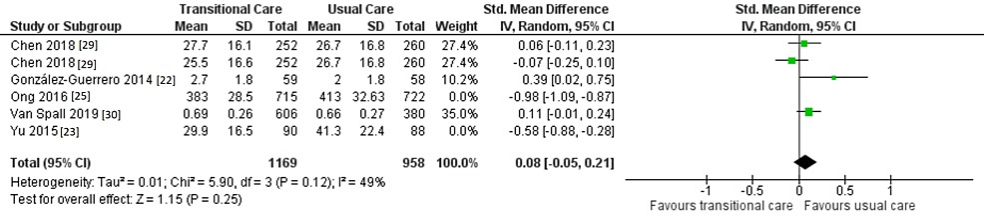
Sensitivity analysis for TCIs' effect on QoL. CI: confidence interval; IV: inverse variance; SD: standard deviation; TCI: transitional care intervention; QoL: quality of life.

Because of high heterogeneity across the included trials (I^2 ^= 98%), the meta-analysis's findings should be interpreted with caution. Clinically, interpretations of heterogeneity include various TCIs compared, differences in the timing of the QoL evaluation, and variable scales used to assess QoL. As a result, a sensitivity analysis was carried out, as illustrated in Figure 7, and RCTs that are considerably heterogeneous were removed from the analysis [23,25]; these eliminated studies were Ong et al.'s and Yu et al.'s. Results from the sensitivity analysis matched those from the meta-analysis. It was found that participants in the control and intervention groups did not significantly vary in terms of their QoL (p = 0.25).

Discussion

A comprehensive review and meta-analysis encompassing 13 RCTs was done to investigate the effectiveness of TCIs for HF patients. In general, it can be said that TCIs were successful in lowering mortality (moderate SOE), ED visits (low SOE), and hospital readmissions (moderate SOE). However, they were ineffective in enhancing the QoL (very low SOE). Summary of findings and SOE have been explained in in Table 3. 

**Table 3:** Summary of findings table and grade of evidence for reported outcomes CI: confidence interval; RR: risk ratio; ED: emergency department; QoL: quality of life; SMD: standard mean difference

Certainty assessment							Number of patients		Effect		Certainty	
Studies	Study design	Risk of bias	Inconsistency	Indirectness	Imprecision	Other considerations	Transitional care intervention	Usual care	Relative (95% CI)	Absolute (95% CI)		
Hospital readmission												
9	Randomized trials	Serious	Not serious	Not serious	Not serious	None	1595/5664 (28.2%)	1891/6218 (30.4%)	RR 0.82 (0.73 to 0.92)	55 fewer per 1,000 (from 82 fewer to 24 fewer)	⨁⨁⨁◯ Moderate	
Mortality												
10	Randomized trials	Serious	Not serious	Not serious	Not serious	None	420/4530 (9.3%)	531/4796 (11.1%)	RR 0.79 (0.67 to 0.93)	23 fewer per 1000 (from 37 fewer to 8 fewer)	⨁⨁⨁◯ Moderate	
ED visit												
2	Randomized trials	Serious	Not serious	Not serious	Serious	None	401/2898 (13.8%)	605/3470 (17.4%)	RR 0.73 (0.53 to 1.00)	47 fewer per 1000 (from 82 fewer to 0 fewer)	⨁⨁◯◯ Low	
QoL												
5	Randomized trials	Serious	Serious	Not serious	Serious	None	1974	1768	-	SMD 0.18 lower (0.65 lower to 0.29 higher)	⨁◯◯◯ Very low	

The review showed that telephone-based interventions were successful in lowering hospital readmissions within the period after 30 days of discharge. The results were in agreement with previous research findings [15]. Moreover, the majority of the interventions examined in the review included telephone support for following up with patients post-discharge, which is supported by the literature and would result in a 44% reduction in the need for re-hospitalization [35]. This is attributable to increased self-efficacy in managing and adhering to self-care behaviors, lifestyle modifications, and medications, which were enhanced by telephone support [36]. Among the included trials, there were variations in the timing of the phone calls. However, no evidence was identified in the literature regarding the ideal timing and length of telephone follow-up. The use of telephone-based interventions carries significant benefits as it overcomes the impracticality of frequent clinic visits by feeble HF patients and is more cost-effective than usual care with an incremental ratio of €8,270 [37,38].

Surprisingly, mortality evaluated 30 days after discharge was unaffected by telephone follow-up. The delay in the administration of phone calls provides a possible explanation, as it was conducted within 30 days of discharge [29] and at 12 weeks after discharge [31]. However, it was found that there was a reduction in the overall mortality following the administration of a clinic-based intervention in conjunction with early telephone follow-up conducted within 48 hours of discharge [22]. Therefore, it is believed that telephone follow-up was best done between 24 and 72 hours following discharge.

The meta-analysis revealed debatable results about the efficacy of telemonitoring intervention even though the number of RCTs examining its effectiveness is increasing. The review showed no effect on mortality and rehospitalizations beyond 30 days after discharge. However, a reduction in ED visits and deaths in the first 30 days following discharge was noted. This can be attributed to the fact that HF patients are more prone to deterioration in the first month following discharge [39], which may be related to a lack of discharge optimization, and a lack of proper education leading to poor adherence and subsequent deterioration. A telemonitoring system might be useful in recognizing early symptoms of deterioration and warranting medical review and lowering mortality within the first 30 days as a result.

In addition to the above-mentioned advantages, telemonitoring has its own disadvantages. Despite strategies arranged to encourage adherence to the intervention, only half of the participants consistently entered their data into the system [25]. Additionally, its effectiveness can be hindered by technical problems which required professional assistance. Moreover, the cost of implementing such an intervention is expected to be significant. However, literature suggested that telemonitoring was more cost-effective than usual care [38].

Hospital readmission and mortality did not appear to be affected by the educational intervention. This finding is controversial as evidence in the literature reported a reduction in rehospitalization after implementing such interventions [39]. Although Van Spall and colleagues [30] used a strict methodology, their findings agreed with those of the meta-analysis. This is because the majority of the participants were older people with multiple comorbidities, which would hinder their ability to comply with the suggested intervention. Moreover, the experiment was also carried out at facilities that received financing and incentives to lower HF rehospitalizations, which encouraged them to provide more baseline standard care and, thus, diluted the impact of the intervention. In contrast, Boyde et al. [26] and Kazemi Majd et al. [33] discovered that educational interventions were successful in lowering hospital readmissions beyond 30 days following release. This can be attributable to the one-year follow-up duration utilized in the interventions which is an approach that is supported by the literature [40].

It was found that HF readmissions can be decreased more effectively with high-intensity TCIs, which involve multidisciplinary teams, repeated coaching, and variable method of communication [15]. According to the statistical analysis, high-intensity multicomponent intervention is successful in lowering hospital readmissions within and after 30 days of the index hospitalization. However, due to a lack of cost-effective analysis, the application of multicomponent interventions may be questionable.

Studies in the literature indicated that the application of TCI improved QoL among HF patients [19]. However, the meta-analysis showed no gain in QoL. Due to significant heterogeneity among RCTs included in the analysis, this result should be regarded with caution. Nevertheless, TCIs enhance self-care behaviors [29]. For example, those who underwent TCIs measured their weight on a regular basis, and this was linked to better health outcomes in HF patients [29].

Strength and Limitations

This systematic review was carried out with rigorous methodology. Meta-analysis improves the generalizability of the finding by calculating the impact magnitude. In addition, only RCTs, which have a higher hierarchy of evidence, were included in the review. Furthermore, bias within included RCTs was extensively assessed with an evidence-based tool. Additionally, the overall grade of the evidence was evaluated, which would boost the review findings' credibility.

On the other hand, the review has some limitations. The findings of the review might not be generalizable due to the limited number of studies included. In addition, since almost all included RCTs combined at least two interventions, it was difficult to guarantee the efficacy of the sole intervention. Additionally, the HF-specific mortality and readmissions were not evaluated. As a result, mortality and readmission from causes other than HF may have an impact on review results.

Although the risk of bias was assessed, most of the included studies expressed "some concern." As a result, confidence in the findings, and the strength of the evidence, was lowered. Additionally, due to extreme heterogeneity among the included studies, the SOE for QoL outcome was very low.

## Conclusions

Given the controversy surrounding TCIs' efficacy and the significant benefits obtained by their implementation, this is a matter worth being investigated. Therefore, 13 RCTs were reviewed and included in statistical analysis to evaluate the effectiveness of TCIs.

It was found that introducing TCI would lower all-cause mortality and readmission rates. TCIs decrease ED visits, yet it is debatable if they enhance the QoL. Specifically, telephone base support was found to be the most effective in reducing rehospitalization. When paired with additional interventions, such as clinic visits, telephone support interventions were found to be effective in reducing mortality. Telemonitoring has also been demonstrated to be useful in monitoring and medically optimizing patients throughout the care gap as well as helping patients during their most vulnerable time right after discharge.
